# A Facile High-Throughput Model of Surface-Independent Staphylococcus aureus Biofilms by Spontaneous Aggregation

**DOI:** 10.1128/mSphere.00186-21

**Published:** 2021-04-28

**Authors:** Terrence Cheng, Nelson S. Torres, Ping Chen, Anand Srinivasan, Sandra Cardona, Grace C. Lee, Kai P. Leung, Jose L. Lopez-Ribot, Anand K. Ramasubramanian

**Affiliations:** a Department of Chemical and Materials Engineering, San José State University, San José, California, USA; b Department of Biology, The University of Texas at San Antonio, San Antonio, Texas, USA; c South Texas Center for Emerging Infectious Diseases, The University of Texas at San Antonio, San Antonio, Texas, USA; d Division of Combat Wound Repair, U.S. Army Institute of Surgical Research, Fort Sam Houston, Texas, USA; e College of Pharmacy, The University of Texas at Austin, Austin, Texas, USA; f BioBridge Global, San Antonio, Texas, USA; University of Georgia

**Keywords:** MRSA, biofilms, high throughput

## Abstract

Many microbes in their natural habitats are found in biofilm ecosystems attached to surfaces and not as free-floating (planktonic) organisms. Furthermore, it is estimated that nearly 80% of human infections are associated with biofilms. Biofilms are traditionally defined as three-dimensional, structured microbial communities that are attached to a surface and encased in a matrix of exopolymeric material. While this view of biofilm largely arises from *in vitro* studies under static or flow conditions, *in vivo* observations have indicated that this view of biofilms is essentially true only for foreign-body infections on catheters or implants where biofilms are attached to the biomaterial. In mucosal infections such as chronic wounds or cystic fibrosis or joint infections, biofilms can be found unattached to a surface and as three-dimensional aggregates. In this work, we describe a high-throughput model of aggregate biofilms of methicillin-resistant Staphylococcus aureus (MRSA) using 96-well plate hanging-drop technology. We show that MRSA forms surface-independent biofilms, distinct from surface-attached biofilms, that are rich in exopolymeric proteins, polysaccharides, and extracellular DNA (eDNA), express biofilm-related genes, and exhibit heightened antibiotic resistance. We also show that the surface-independent biofilms of clinical isolates of MRSA from cystic fibrosis and central catheter-related infections demonstrate morphological differences. Overall, our results show that biofilms can form by spontaneous aggregation without attachment to a surface, and this new *in vitro* system can model surface-independent biofilms that may more closely mimic the corresponding physiological niche during infection.

**IMPORTANCE** The canonical model of biofilm formation begins with the attachment and growth of microbial cells on a surface. While these *in vitro* models reasonably mimic biofilms formed on foreign bodies such as catheters and implants, this is not the case for biofilms formed in cystic fibrosis and chronic wound infections, which appear to present as aggregates not attached to a surface. The hanging-drop model of biofilms of methicillin-resistant Staphylococcus aureus (MRSA), the major causative organism of skin and soft tissue infections, shows that these biofilms display morphological and antibiotic response patterns that are distinct from those of their surface-attached counterparts, and biofilm growth is consistent with their *in vivo* location. The simplicity and throughput of this model enable adoption to investigate other single or polymicrobial biofilms in a physiologically relevant setting.

## Abstract (precis)

The canonical model of biofilm formation begins with the attachment and growth of microbial cells on a surface. While these *in vitro* models reasonably mimic biofilms formed on foreign bodies such as catheters and implants, this is not the case for biofilms formed in cystic fibrosis and chronic wound infections, which appear to present as aggregates not attached to a surface.

## INTRODUCTION

Most of our knowledge of medical biofilms arises from the experience with biofilm formation on foreign bodies such as implants or catheters and *in vitro* models of the *in vivo* physiology using static or flow-based systems (1). Biofilms have traditionally been defined as structured microbial communities that are “attached to a surface” and encased in a matrix of exopolymeric material variously composed of polysaccharides, extracellular DNA (eDNA), and proteins (2, 3). This canonical model of the biofilm life cycle begins with the adhesion of cells to the surface, followed by proliferation and the production of exopolymeric material characteristic of biofilms. As the biofilm reaches maturity, to complete the cycle, the cells are dispersed for reseeding at distant sites (4).

However, infrequent but careful studies in the past 15 to 20 years have reported that *in vivo* biofilms considerably depart from the *in vitro* canonical surface-attached model in that in certain clinical settings, these biofilms are found as unattached small aggregates (50 to 100 μm) and typically dispersed in mucus or compromised soft tissue (5, 6). Aggregates of Pseudomonas aeruginosa, Staphylococcus aureus, Escherichia coli, Staphylococcus lugdunensis, and Mycobacterium abscessus with increased tolerance to antibiotics and embedded in a biofilm matrix have been isolated from the lungs of cystic fibrosis patients; chronic wounds; and middle ear, infectious arthritis, and periprosthetic joint infections (7–10). These aggregates of cells show active group behavior rather than just representing a passive collection of individual cells: they demonstrate phenotypic attributes of biofilms, including the presence of an encasing matrix, antibiotic tolerance, and host immune evasion. These observations demand a paradigm shift in the definition of biofilms and biomimetic models of biofilm physiology.

Several *in vitro* approaches within the past decade have extended the definition of biofilm beyond the canonical model of surface attachment. It has been shown that S. aureus or P. aeruginosa bacteria encapsulated in agarose or alginate beads show phenotypic characteristics of biofilm infections, including the formation of dense aggregates, reduced growth rates, local hypoxia, and antibiotic tolerance (11, 12). Spontaneous aggregation driven by low-shear conditions either in a shake flask or by magnetic levitation also produces biofilm-like morphological and drug susceptibility characteristics (13, 14). We have shown that biofilms of single and polymicrobial nanocultures of Candida albicans, S. aureus, and P. aeruginosa encapsulated in alginate or collagen grow as dense microcolonies, express copious exopolymeric matrix, and show increased antibiotic resistance (15–17). Our nanoculture microarray platform not only facilitated ultrahigh-throughput screening of antibiofilm drugs but also prompted insights into the possibility of biofilm formation in three dimensions instead of traditional, gravity-driven surface attachment in two dimensions as a prerequisite first step. One of the major limitations of these *in vitro* models is that they fail to capture the *in vivo* physiology of freestanding biofilms as they require extraneous drivers of aggregation such as binding proteins or encapsulating matrix or may not be easily adaptable to standard laboratory procedures for convenient high-throughput screening for antimicrobial susceptibility.

In this study, we describe the development of an alternative model that recapitulates biofilm formation by methicillin-resistant S. aureus (MRSA) driven by bacterial aggregation in the absence of attachment to a solid substrate or an encapsulating matrix. Our approach to developing this model was inspired by the three-dimensional (3D) tumor spheroids formed from cancer cell suspensions using hanging-drop culture (18, 19). This model uses a 96-well template, which is convenient for adoption to high-throughput assays on standard laboratory platforms. The biofilm-producing capacity and antimicrobial resistance of MRSA pose a major therapeutic challenge in diseases ranging from mild skin and soft tissue infections to life-threatening cases of pneumonia and endocarditis (20). We demonstrate that MRSA forms robust biofilm aggregates in the hanging drops, confirmed by phenotypic and genotypic evidence, and that the clinical isolates from certain anatomical locations prefer growth as aggregate biofilms, consistent with their physiological relevance.

## RESULTS

### Development of hanging-drop biofilms.

**FIG 1:**
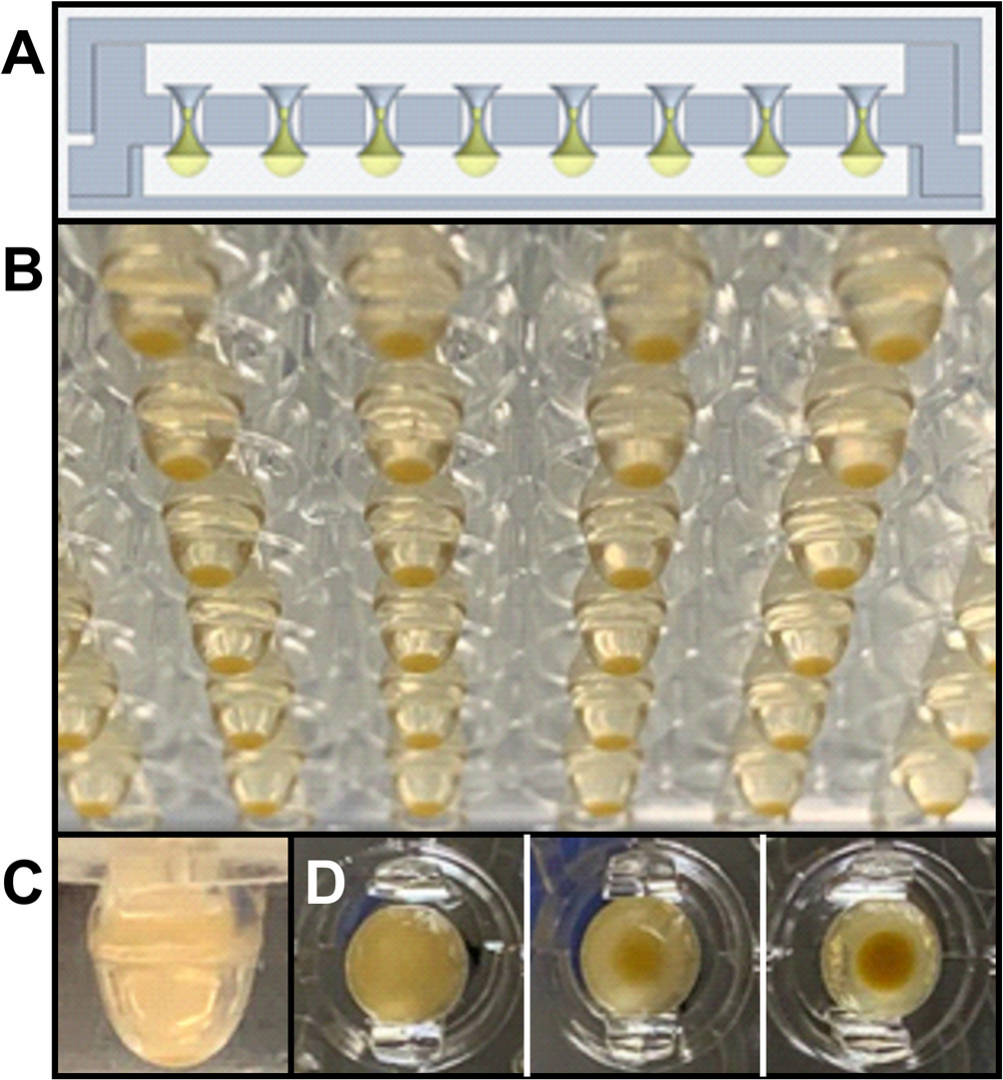
High-throughput hanging-drop system for S. aureus biofilms. (A) Schematic of the 96-well hanging-drop-plate system; (B) bottom view of hanging drops; (C) side view of a single 50-μl hanging drop; (D) top view of hanging drops during the course of biofilm development.

We utilize GravityPLUS 96-well plates (InSphero Inc., ME), which consist of three separate pieces: a lid, a gravity tray, and the hanging-drop plate (Fig. 1A). The tray and the hanging-drop plates have built-in water reservoirs that run along the perimeter and help minimize the loss of drop volume due to evaporation during incubation at 37°C. Fifty microliters of methicillin-resistant S. aureus strain TCH1516 in tryptic soy broth (TSB) medium at an optical density at 600 nm (OD_600_) of 0.01 was placed into open wells as hanging drops (Fig. 1B). We typically observed that the initially turbid drop forms two phases as cells settle down and aggregate at the bottom of the drop (Fig. 1C and D). We used medium containing only TSB without any additives commonly used in adherent biofilm cultures (fibrinogen, serum, or additional glucose) (21, 22). We examined the growth of biofilms by monitoring the fluorescence of SYTO 9 and SYPRO ruby stains, which bind to intracellular nucleic acids and exopolymeric proteins, respectively. We initially evaluated biofilm growth after 24 h of cell seeding at various cell seeding densities. Based on these initial microscopic observations on cell number and biofilm matrix, a seeding density corresponding to an OD_600_ of 0.01 was found to be optimal for biofilm growth and was chosen for all subsequent experiments. Interestingly, at this seeding density, biofilms could be visualized in well plates only when the TSB medium contained serum but not in TSB-only medium because the initiation of biofilm formation is mediated by cell adhesion to serum proteins adsorbed on the surface (data not shown).

### Characterization of hanging-drop biofilms.

**FIG 2:**
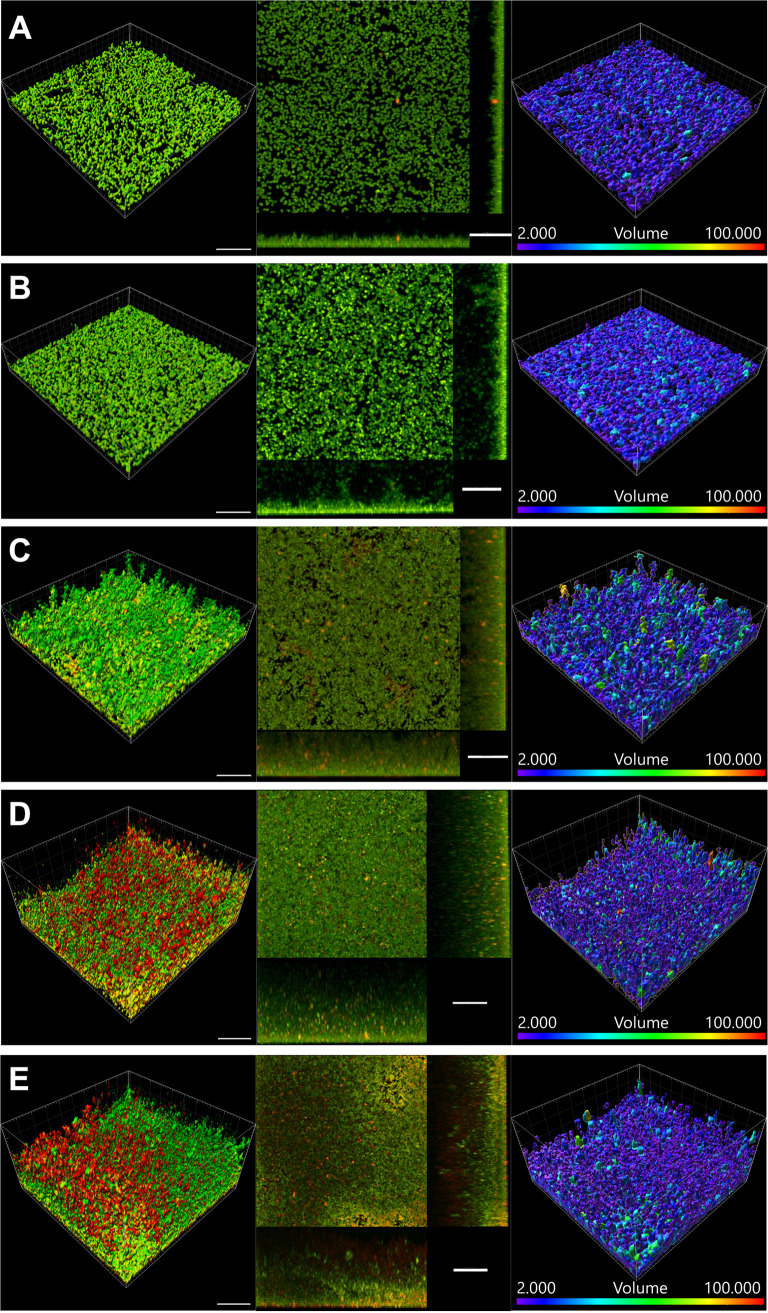
Evolution of hanging-drop biofilms. Hanging-drop cultures of S. aureus stained with SYTO 9 (green) and SYPRO ruby (red) and imaged *in situ* using confocal laser scanning microscopy at the indicated time points show increases in biofilm thickness and microcolony size. Shown are representative 3D, *x-y*, *y-z*, and *x-z* section views and microcolony volumes at 3, 6, 12, 24, and 48 h postseeding. Bars, 20 μm. The volume range is 2 to 100 μm^3^.

We used confocal microscopy to examine the biofilms in their original conformation as a hanging drop and monitored development at 3, 6, 12, 24, and 48 h postseeding, without fixation and gently transferring the biofilms to a coverslip. We observed that biofilm formation begins from single cells (green fluorescence), and the biofilm aggregate increases over the period of observation (Fig. 2A to E; see also Movies S1 and S2 in the supplemental material). The biofilm protein content (red fluorescence) also increased during this time. The sectional views over 100 by 100 μm^2^ show the increase in thickness over time and that the cells are nearly uniformly distributed throughout the thickness of the biofilm. Furthermore, closer inspection of the biofilms indicates that the biofilm is composed of S. aureus cells present as microcolonies, which grow in size during the observation period, with a majority of the colonies having a <20-μm^3^ volume.

10.1128/mSphere.00186-21.5 MOVIE S1 3D visualization of hanging-drop biofilms after 24 h of growth, stained with SYTO 9 (green) and SYPRO ruby (red). Download Movie S1, AVI file, 17.2 MB. Copyright © 2021 Cheng et al. 2021 Cheng et al. https://creativecommons.org/licenses/by/4.0/ This content is distributed under the terms of the Creative Commons Attribution 4.0 International license.

10.1128/mSphere.00186-21.6 MOVIE S2 3D visualization of hanging-drop biofilms after 24 h of growth, stained with SYTO 9 (green), WGA (red), and DAPI (blue). Download Movie S2, AVI file, 17.2 MB. Copyright © 2021 Cheng et al. 2021 Cheng et al. https://creativecommons.org/licenses/by/4.0/ This content is distributed under the terms of the Creative Commons Attribution 4.0 International license.

**FIG 3:**
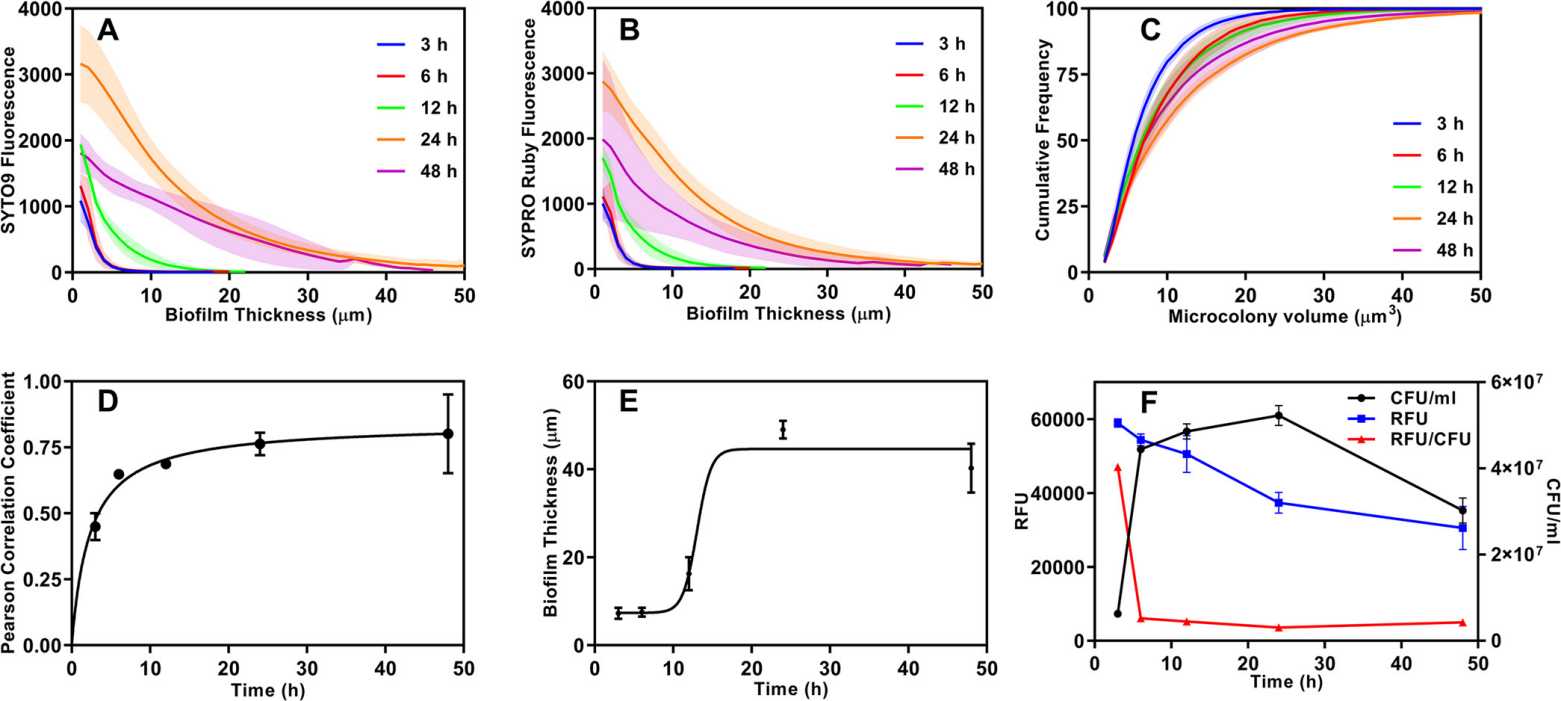
Quantification of biofilm development. Hanging-drop biofilms were stained with SYTO 9 and SYPRO ruby at the indicated time points, and the fluorescence intensities from confocal images were quantified. (A and B) Distribution of cells (A) and exopolymeric matrix (B) from the bottom to the top of the biofilm at the indicated time points. (C) Distribution of the sizes of individual microcolonies within biofilms. (D) Three-dimensional Pearson correlation coefficient for colocalization of cells and matrix within the biofilm. (E) Biofilm thickness quantified as the range over which fluorescence intensities dropped to negligible levels. The results are presented as means ± standard deviations (SD) (*n *= 4). (F) Metabolic activity and viability of cells in the biofilms estimated by the change in fluorescence at 530/590 nm (excitation/emission) using a PrestoBlue assay and colony counting, respectively. RFU/CFU values were scaled up by a factor of 5 × 10^6^ for graphical representation on the RFU axis. The results are expressed as means ± standard errors of the means (SEM) (*n *= 3).

We used the fluorescence intensities from cells and biofilm matrix at various depths shown in Fig. 2 to quantify the kinetics of biofilm formation (Fig. 3). Based on the fluorescence intensities, we observed that both cell and matrix densities were maximum at the bottom of the aggregate. The first 6 h showed only a slow increase, but between 12 and 24 h, the biofilm expanded significantly, and between 24 and 48 h, a decrease in the intensities was noticeable (Fig. 3A and B). This decrease in intensity at longer times may indicate dispersion or endogenous enzymatic degradation of the matrix or a loss of cell viability. We quantified the volume of microcolonies within the biofilm, which is shown in Fig. 2. Expectedly, the volume of the bacterial microcolonies increased with biofilm growth (Fig. 3C): during early stages of growth (3 h), the majority (75%) of the microcolonies were <10 μm^3^, and by 24 h of growth, this value increased to 20 μm^3^, indicating an increase in cell number within these colonies. As a comparison, we visualized the MRSA biofilms grown as traditional adherent cultures on surfaces (Fig. S1 and Movies S3 and S4). Even on surfaces coated with collagen, biofilms did not form when only TSB was used, and the medium had to be supplemented with serum to observe biofilm growth. The architecture of adherent biofilms is substantially different from that of the hanging-drop biofilms, with disjointed but more cohesive microcolonies with sizes comparable to those of the hanging biofilms (Fig. S1A and C).

10.1128/mSphere.00186-21.1 FIG S1 Architecture of surface-attached biofilms after 24 h of growth. (A and B) Confocal microscopy images of biofilms stained with SYTO 9 (green) and SYPRO ruby (red) (A) or SYTO 9 (green), WGA (red), and DAPI (blue) (B). Bar, 20 μm. The volume range is 2 to 100 μm^3^. (C) Quantification of microcolony volume presented as the mean ± SD (*n *= 5). Download FIG S1, TIF file, 0.8 MB. Copyright © 2021 Cheng et al. 2021 Cheng et al. https://creativecommons.org/licenses/by/4.0/ This content is distributed under the terms of the Creative Commons Attribution 4.0 International license.

10.1128/mSphere.00186-21.7 MOVIE S3 3D visualization of surface-attached biofilms after 24 h of growth, stained with SYTO 9 (green) and SYPRO ruby (red). Download Movie S3, AVI file, 17.2 MB. Copyright © 2021 Cheng et al. 2021 Cheng et al. https://creativecommons.org/licenses/by/4.0/ This content is distributed under the terms of the Creative Commons Attribution 4.0 International license.

10.1128/mSphere.00186-21.8 MOVIE S4 3D visualization of surface-attached biofilms after 24 h of growth, stained with SYTO 9 (green), WGA (red), and DAPI (blue). Download Movie S4, AVI file, 17.2 MB. Copyright © 2021 Cheng et al. 2021 Cheng et al. https://creativecommons.org/licenses/by/4.0/ This content is distributed under the terms of the Creative Commons Attribution 4.0 International license.

We used the three-dimensional Pearson correlation coefficient (PCC) to quantify the colocalization of biofilm matrix and cells (Fig. 3D). PCC estimates the localization of red and green intensities in any given pixel and varies between 0 and 1, corresponding to no correlation and a perfect correlation between the two colors, respectively (23). Our data show that except during the initial development phase (3 h) when the matrix density was lower, the cells and proteinaceous matrix are in close association from 6 h to 48 h. The biofilm thickness was quantified as the distance spanning the maximum to minimum fluorescence intensities, i.e., lower than the nominal relative fluorescence intensity of 50 U. The biofilm thickness increased from 5 μm to 50 to 60 μm over the observation period, with dramatic growth being observed between 12 h and 24 h (Fig. 3E). Since it appears that the biofilm thickness attained saturation, and the matrix density decreased by 24 h, we estimated the metabolic activity using a PrestoBlue assay and the viable CFU to capture the state of the cells in the biofilm (Fig. 3F). The metabolic activity (relative fluorescence units [RFU]) quickly accelerated by 6 h and remained high until 12 to 24 h, beyond which the activity decreased modestly. The viability data (CFU per milliliter) indicated that the cells grew rapidly in the first 3 h to 6 h, beyond which the viable cell count remained constant until 24 h, after which the viability decreased by 48 h. Based on these two assays, we estimated that the specific metabolic activity per viable cell (RFU per CFU per milliliter) dropped drastically by 6 h but remained nearly constant until 24 h, with a modest decrease thereafter, suggesting that the biofilms are fully matured by 24 h.

**FIG 4:**
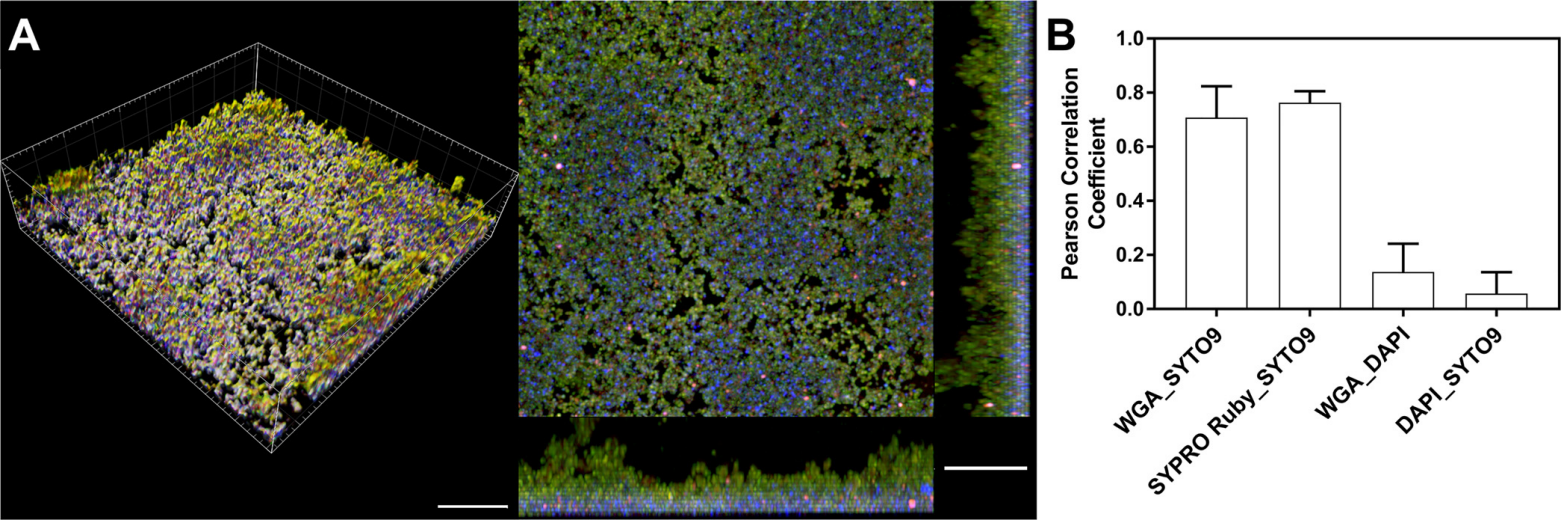
Distribution of biofilm matrix components at 24 h of growth. Hanging-drop cultures of S. aureus after 24 h were stained for nuclear DNA, PNAG, and eDNA with SYTO 9 (green), WGA (purple), and DAPI (blue), respectively, and imaged *in situ* using confocal laser scanning microscopy. (A) Representative 3D and section views. Bars, 20 μm. (B) 3D Pearson’s correlation coefficient estimates of the colocalization of various components within the biofilm matrix, which show that PNAG and matrix protein, but not eDNA, are colocalized with cells. The results are expressed as means ± SD (*n *= 4).

In addition, to evaluate the presence of other exopolymeric matrix components, i.e., extracellular DNA (eDNA) and poly-*N*-acetylglucosamine (PNAG), which acts as an intercellular adhesin, we stained the biofilms with 4′,6-diamidino-2-phenylindole (DAPI) and wheat germ agglutinin (WGA), respectively (Fig. 4). The cells were counterstained with SYTO 9. Copious amounts of eDNA and PNAG were observed in the 24-h biofilms (Fig. 4A). Interestingly, PCC analysis showed that PNAG, but not eDNA, was closely associated with the cells (Fig. 4B). As a comparison, in surface-attached biofilms, the amount of eDNA appeared to be smaller than what was seen in hanging-drop biofilms (Fig. S1B).

### Susceptibility of biofilms to antibiotics.

**FIG 5:**
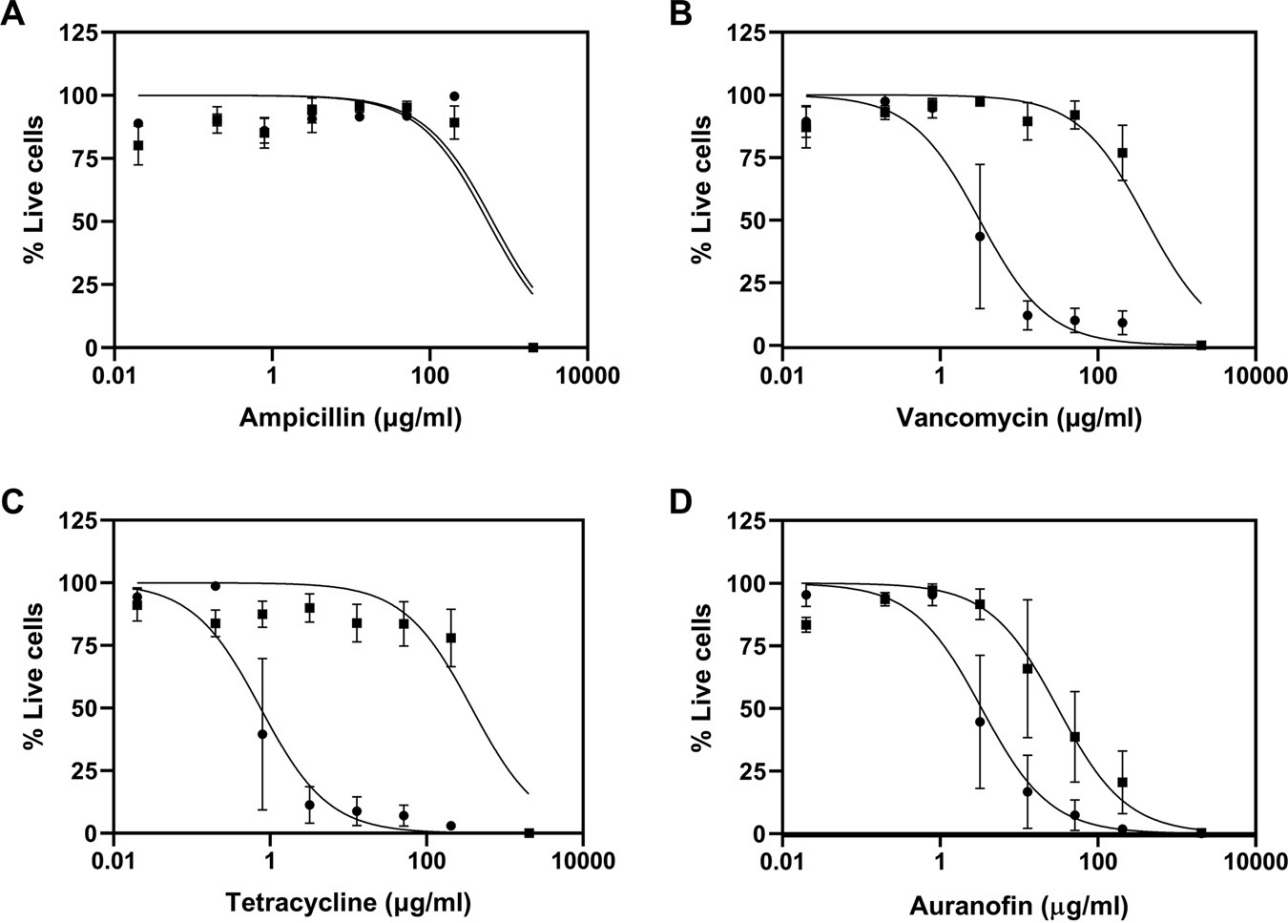
Dose-response profiles of antibiotics in the inhibition of biofilm formation and eradication of preformed biofilms. Hanging-drop cultures were incubated with drugs at the indicated concentrations either at the beginning of 24 h (inhibition of biofilm formation) (circles) or after 24 h of culture and incubation for an additional 24 h (eradication of preformed biofilms) (squares). The results are expressed as means ± SEM (*n *= 3).

One of the distinguishing characteristics of cells within biofilms is their increased resistance to antibiotics. Thus, we evaluated the susceptibility of hanging-drop biofilms to ampicillin, vancomycin, tetracycline, and auranofin against both inhibition of biofilm formation and eradication of preformed biofilms (Fig. 5). As expected, the biofilms were resistant to ampicillin since the MRSA strains are resistant to β-lactam antibiotics (Fig. 5A). On the other hand, both vancomycin and tetracycline inhibited biofilm formation with a 50% inhibitory concentration (IC_50_) in the 1- to 3-μg/ml range but were quite ineffective in eradicating preformed biofilms (Table 1). We observed that auranofin, a repurposed antirheumatic, was effective in inhibiting biofilm formation at 3 μg/ml and against preformed biofilms at a concentration of 56 μg/ml. We also compared the susceptibilities of surface-attached biofilms to these drugs (Table 1; Fig. S2). The surface-attached biofilms, as expected, were also resistant to ampicillin. The inhibition of surface-attached biofilms by vancomycin, tetracycline, and auranofin occurred at concentrations of 0.3 to 1.6 μg/ml, which is comparable to the hanging-drop biofilms. The preformed surface-attached biofilms were indeed resistant to vancomycin and tetracycline with IC_50_s in the 100-μg/ml range but susceptible to auranofin (∼8 μg/ml). Auranofin was highly effective against preformed surface-attached biofilms, consistent with the remarkable efficacy of auranofin reported in previous studies on surface-attached S. aureus and P. aeruginosa biofilms (24, 25). However, the lower IC_50_ values of surface-attached biofilms than of hanging-drop biofilms indicate that the preformed hanging-drop biofilms are more resistant to these different classes of drugs than surface-attached biofilms.

**TABLE 1:** IC_50_s of hanging-drop biofilm and surface-attached biofilm cultures of S. aureus

Drug	Mean IC_50_ (μg/ml) ± SEM (*n *= 3)			
	Hanging-drop biofilms		Surface-attached biofilms	
	Inhibition	Eradication	Inhibition	Eradication
Ampicillin	551.33 ± 42.77	517.03 ± 55.32	491.63 ± 39.98	581.07 ± 31.54
Vancomycin	3.32 ± 1.24	342.33 ± 113.29	1.60 ± 0.17	251.35 ± 110.58
Tetracycline	1.08 ± 0.66	303.83 ± 147.90	0.34 ± 0.01	124.29 ± 24.11
Auranofin	3.08 ± 1.25	56.25 ± 26.92	0.81 ± 0.29	7.52 ± 2.81

10.1128/mSphere.00186-21.2 FIG S2 Dose-response profiles of antibiotics in the inhibition of biofilm formation and eradication of preformed biofilms in surface-attached biofilm cultures. Biofilm cultures were incubated with drugs at the indicated concentrations either at the beginning of 24 h (inhibition of biofilm formation) (circles) or after 24 h of culture and incubation for an additional 24 h (eradication of preformed biofilms) (squares). The results are expressed as means ± SEM (*n *= 3). Download FIG S2, TIF file, 1.5 MB. Copyright © 2021 Cheng et al. 2021 Cheng et al. https://creativecommons.org/licenses/by/4.0/ This content is distributed under the terms of the Creative Commons Attribution 4.0 International license.

### Upregulation of biofilm-related genes.

**FIG 6:**
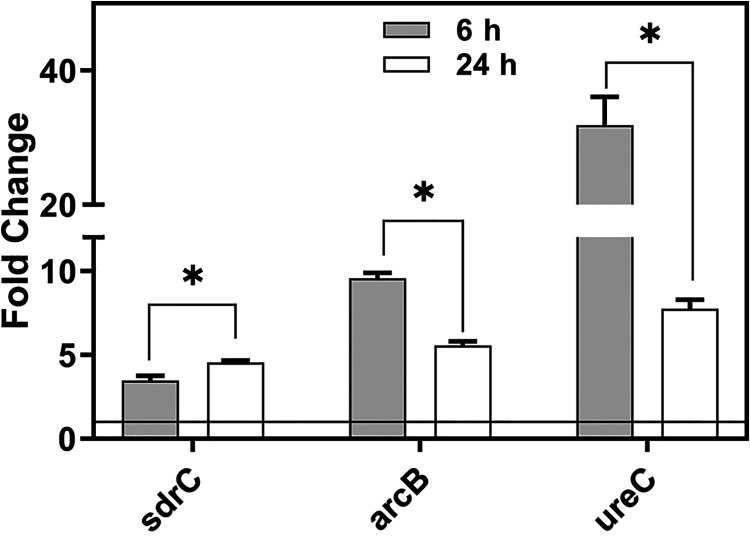
Upregulation of gene expression levels in hanging-drop biofilms. All genes in biofilms are significantly overexpressed compared to baseline expression levels in mid-log-phase planktonic cells, represented as a line at 1.0. The results are expressed as means ± SEM (*n *= 3) (*, *P < *0.05).

Having performed the phenotypic characterization of hanging-drop biofilms, we sought to evaluate the transcriptomic changes associated with biofilm growth. To this end, we used the QuantiGene Plex assay to quantify the expression of three representative genes that are known to be upregulated in surface-attached S. aureus biofilms: *sdrC* (Ser-Asp-Arg-rich fibrinogen-binding protein), *arcB* (ornithine transcarbamylase), and *ureC* (urease accessory protein C) (26–29). These genes, *sdrC*, *arcB*, and *ureC*, are key to encoding proteins for fibrinogen-mediated cell adhesion, extraction, and catabolism of arginine and metabolism of urea in establishing an ecological niche, respectively. Comparison of the expression levels of these genes in the hanging-drop biofilms with those in planktonic cultures revealed that these genes are differentially upregulated at both early (6-h) and late (24-h) stages of biofilm growth (Fig. 6). The expression levels decreased between 6 h and 24 h, suggesting that optimal biofilm growth was reached between these two time intervals, consistent with the metabolic activity data. Furthermore, we notice that while *ureC* and *arcB* levels increased nearly 30- and 10-fold, respectively, at 6 h, *sdrC* increased only 3-fold in hanging-drop biofilms at both 6 h and 24 h. In contrast, the expression levels of these three genes in surface-attached biofilms were not statistically different from those in planktonic cultures at 6 h but increased dramatically by 24 h, indicating that biofilm maturation occurs at a later stage than in hanging-drop biofilms (Fig. S3). Of note, while *sdrC* levels were unchanged at 6 h and 24 h in hanging-drop biofilms, *sdrC* levels increased from insignificant levels at 6 h to nearly 35-fold by 24 h, suggesting that unlike surface-attached biofilms, the hanging-drop biofilms may not depend on fibrinogen-mediated adhesion for biofilm formation.

10.1128/mSphere.00186-21.3 FIG S3 Upregulation of gene expression levels in surface-attached biofilms compared to mid-log-phase planktonic cells, represented as a line at 1.0. The expression levels were not statistically different from the baseline at 6 h but increased dramatically by 24 h. The results are expressed as means ± SEM (*n *= 3) (*, *P < *0.05). Download FIG S3, TIF file, 0.3 MB. Copyright © 2021 Cheng et al. 2021 Cheng et al. https://creativecommons.org/licenses/by/4.0/ This content is distributed under the terms of the Creative Commons Attribution 4.0 International license.

### Biofilms of MRSA clinical isolates.

In hanging-drop and surface-attached modes, we formed biofilms from 10 community-associated MRSA (CA-MRSA) clinical isolates that were collected from patients from various anatomical locations, namely, sputum, skin, bone, nares, and blood, and diseases, namely, cystic fibrosis, wound, and central catheter-related infections. The laboratory strain used in this study, S. aureus TCH1516, was used for comparison. As shown in Fig. S4, the cystic fibrosis strain P1V44 demonstrated the most metabolic activity in the hanging-drop mode, while the central catheter strain HH10 demonstrated the most growth in the surface-attached mode.

10.1128/mSphere.00186-21.4 FIG S4 Clinical isolates of MRSA after 24 h of culture as hanging-drop biofilms and surface-attached biofilms. (A) Metabolic activity in hanging-drop biofilms; (B) biofilm density in surface-attached biofilms. The results are expressed as means ± SD for six replicate biofilms from one representative experiment, and the results were consistent across two independent runs (*, *P < *0.01). Download FIG S4, TIF file, 1.2 MB. Copyright © 2021 Cheng et al. 2021 Cheng et al. https://creativecommons.org/licenses/by/4.0/ This content is distributed under the terms of the Creative Commons Attribution 4.0 International license.

**FIG 7:**
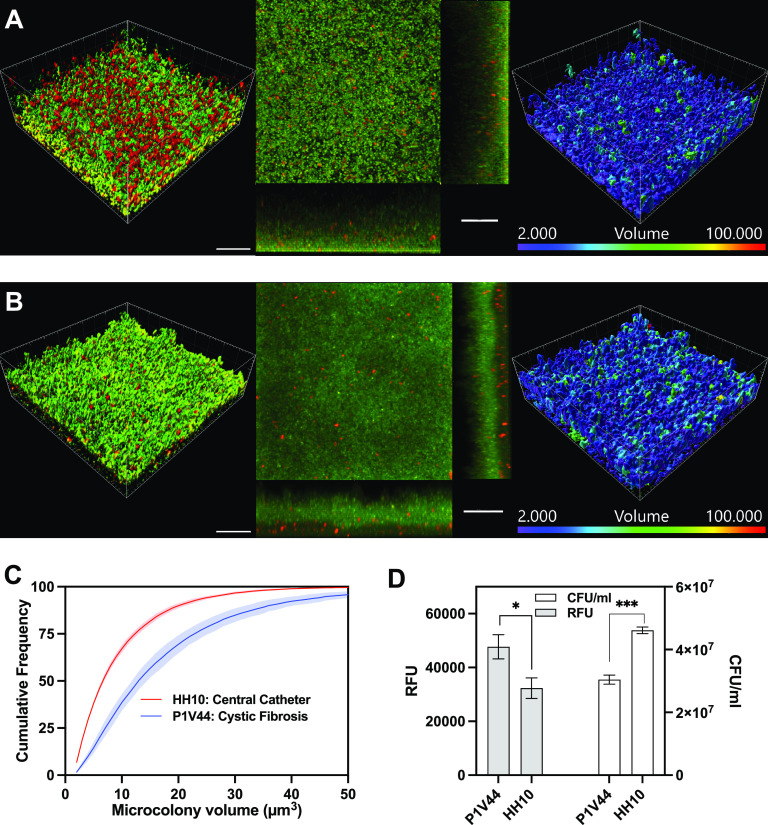
(A and B) Confocal microscopy visualization of hanging-drop biofilm cultures at 24 h of growth of MRSA isolates from infections related to a central catheter, HH10 (A), and cystic fibrosis, P1V44 (B). Bars, 20 μm. The volume range is 2 to 100 μm^3^. (C) Distribution of microcolony volumes within the biofilms. (D) Metabolic activity and viability at 24 h of growth. The results are expressed as means ± SEM (*n *= 3) (*, *P < *0.05; ***, *P < *0.001).

We paid closer attention to these two isolates, i.e., isolates from cystic fibrosis (P1V44) and central catheter-related (HH10) infections, and of note, the former is not associated with an abiotic surface, while the latter is attached to a surface. We observed qualitatively clear differences between the isolates: the biofilms from the cystic fibrosis isolate were less rich in the matrix than the isolate from central catheter infection (Fig. 7A and B). The quantification of cell density clearly revealed the presence of larger microcolonies in cystic fibrosis biofilms than in central catheter-related infection: the majority (75%) of the microcolonies in biofilms of the cystic fibrosis isolate were <22 μm^3^ in volume, but the corresponding maximum volume was 11 μm^3^ in the case of biofilms from central catheter infection (Fig. 7C). Together, these data show that the hanging-drop biofilms may provide a different view of biofilm physiology from that of adherent biofilms. We also observed that the metabolic activity of the P1V44 strain in the hanging-drop biofilms was higher than that of the HH10 strain, although the viable cell count was lower for the P1V44 strain than for the HH10 strain (Fig. 7D). Therefore, the specific metabolic activity estimated per cell of the P1V44 strain is ∼50% higher than that of HH10 in hanging-drop biofilms. As a comparison, in the surface-attached mode, the HH10 strain showed more biofilm growth than the P1V44 strain (respective OD_600_s of 0.036 ± 0.02 versus 0.025 ± 0.02 [*n *= 3] [*P < *0.05]).

## DISCUSSION

In this work, we present a new *in vitro* model of biofilm formation using a hanging-drop technique wherein cell aggregation promotes the formation of biofilms without the need for surface attachment. The resulting biofilms are rich in exopolymeric matrices and are resistant to antibiotic treatment. Furthermore, the high-throughput, well-based format of the model makes it an ideal, new platform for phenotypic characterization and antibiotic susceptibility testing.

Many microbes in their natural habitats are found in biofilm ecosystems and not as individual (planktonic) organisms. Seminal work in the 1980s changed the classical perception of microorganisms as unicellular life forms, which was almost entirely based on the pure-culture mode of growth, to biofilm ecosystems wherein the microbes are attached to surfaces and not as free-floating (planktonic) organisms (3). Since then, the canonical model of biofilm formation has necessitated a surface for cell adhesion and biofilm growth. This concept is replicated in several *in vitro* models that include both static (multiwell plates and culture tubes), and flow (open or closed channel) systems (30, 31). These models faithfully capture biofilms formed on implantable surfaces and catheters but not biofilms found during actual infection in *in vivo* tissues.

Bacterial aggregates (“bioflocs”) have been extensively studied in environmental microbiology, while most attention in medical microbiology has been centered on surface-attached biofilms (32). However, only within the past decade has the role of surface-independent biofilms received attention as causative agents of a number of chronic infections (33, 34). Importantly, these biofilms are not found attached to any biotic or abiotic surfaces but are found as free-floating clusters. Free-floating aggregates of S. aureus with biofilm-like characteristics, including heightened antibiotic resistance and the presence of an exopolymeric matrix, have been isolated from synovial fluid of joint infections (10), chronic wound infections (35), and mucosal secretions in cystic fibrosis infections (36). Clearly, the traditional *in vitro* models of surface-attached biofilms will not be appropriate for understanding CA-MRSA chronic infections in these clinical scenarios.

We have demonstrated that the hanging-drop technique, which is popularly used to model 3D tumor spheroids, can be successfully adapted to study nonattached aggregate biofilms. As gravity drives the proximity between cells, the cells inevitably aggregate, and the cell-cell interactions likely drive biofilm formation. Thus, this system mimics an infection-related environment in restricted spaces of mucosal tissues in alveoli or the urinary tract (20). Of note, the hanging-drop biofilms are fundamentally different from pellicle biofilms (37). Biofilm formation in the hanging drop is a volumetric expansion-and-maturation process initiated by cellular aggregation due to gravitational settling. On the other hand, pellicle formation is a surface phenomenon driven by surface tension forces, often against gravity, at the air-liquid interface. In the majority of cases, pellicle formation begins with the attachment of the bacteria to the solid surface at the interface between the air and liquid (38).

We specifically used medium without any fibrinogen so that we could study biofilm formation as a process distinct from fibrinogen-mediated clumping and agglutination (22). Our transcriptomic and phenotypic data show that biofilm formation is likely not dependent on the interaction with host plasma protein (i.e., fibrinogen) but relies on intercellular interactions and the production of exopolymeric matrix material, including proteins, polysaccharides, and eDNA. The biofilms were observable on the solid substrates only in the presence of fibrinogen in the medium, as eliminating fibrinogen resulted in the complete washout of any surface-attached structures that may have formed in the wells. This suggests an inherent limitation of surface-attached *in vitro* models to study clumping/agglutination-independent biofilms in the absence of exogenously added fibrinogen or serum since the adsorption of fibrinogen or serum is necessary for the initial adhesion of cells to abiotic surfaces.

Consistent with the biofilms from chronic infections, the aggregate biofilms consisted of smaller microcolonies than those of surface-attached biofilms, and the cells were also more uniformly distributed throughout the biofilm volume. Interestingly, we observed clear differences in the growth of hanging-drop biofilms of clinical isolates. The P1V44 strain from cystic fibrosis infection formed hanging-drop biofilms that contained larger microcolonies and significantly higher metabolic activity than the HH10 strain from central catheter infection, although the latter formed stronger biofilms than the P1V44 strain in surface-attached cultures. These observations indicate that the clinical strains prefer their host-like niche in *in vitro* models, and therefore, it is more prudent to use an appropriate model for evaluating antibiotic susceptibility and pathogenicity. Further in-depth characterization with additional clinical isolates is warranted based on the insights presented here.

In summary, we believe that the present study is an important addition to the new wave of methods and experiments that are being developed to better represent the clinical conditions so that the significant gap between *in vitro* predictions and *in vivo* responses, particularly in chronic infections, can be reduced. The high-throughput, 96-well format of the hanging drops can be viewed as a complementary arm to the well plate format for adherent biofilm cultures, which revolutionized the study of antimicrobial susceptibility in the mid-1990s through the mid-2000s (39–41).

## MATERIALS AND METHODS

### Culture conditions.

Methicillin-resistant Staphylococcus aureus (MRSA) strain TCH1516 was purchased from the ATCC. This strain was originally isolated from a pediatric patient with severe sepsis. The clinical isolates used in this study were originally collected from patients in community hospitals and primary care practices affiliated with South Texas (42, 43). All strains used were maintained as frozen stocks by mixing the cell suspension (10^8^ cells per ml) and 50% glycerol in a 1:1 ratio and stored in cryovials at −80°C. To prepare cells for experiments, a pinhead of frozen cells was streaked onto a tryptic soy agar (TSA) plate (BD Bacto; Fisher Scientific, PA). A culture grown overnight was prepared by inoculating 8 ml of tryptic soy broth (TSB) medium (BD Bacto; Fisher Scientific, PA) with a colony from a TSA plate and incubated in an orbital shaker set at 150 rpm at 37°C for 12 h to 16 h. A subculture was then prepared by transferring 500 μl of the culture grown overnight to 20 ml of fresh TSB and incubated at 150 rpm at 37°C until it reached the log phase of growth. The log-phase culture was then washed twice via centrifugation at 4,000 rpm for 15 min and resuspended in phosphate-buffered saline (PBS). After a third centrifugation, the cells were resuspended in fresh TSB and adjusted to a final concentration corresponding to an optical density at 600 nm (OD_600_) of 0.01 using a plate reader (Synergy Neo2; BioTek Instruments, VT). This OD was found to correspond to 10^6^ CFU/ml using a standard curve (44). These clinical isolates were stored and cultured using a procedure similar to the one used for the laboratory strains described above, with one difference: the single colonies from TSA plates were picked and plated two times before expanding the subculture for subsequent experiments.

### Hanging-drop biofilm cultures.

Fifty microliters of the TCH1516 cell suspension was dispensed into the hanging-drop plates (GravityPLUS 96-well plates; InSphero Inc., ME) using extended-tip pipettes. To mitigate evaporation issues, 18 ml of sterile water was placed in the bottom compartment. Three hours, 6 h, 12 h, 24 h, and 48 h after seeding, the hanging drops were analyzed either by microscopy (see below) or for viability. For the latter, 5 μl of PrestoBlue cell viability reagent (Thermo Fisher, MA) was added to each drop, the mixture was incubated for 10 min, and the entire drop was transferred to the wells of a GravityTRAP plate, which is complementary to the GravityPLUS plates. Fluorescence was read at an excitation/emission wavelength of 530/590 nm using a plate reader (Synergy Neo2; BioTek Instruments, VT).

### Confocal fluorescence microscopy.

To visualize and quantify the hanging-drop biofilms, confocal laser scanning microscopy was used. After initial seeding, the hanging drops were incubated at 37°C under static conditions for 3, 6, 12, 24, and 48 h. At each of the given time points, the resulting aggregates were stained with SYTO 9 (Thermo Fisher, MA) at a final concentration of 500 nM for 30 min and with SYPRO ruby (Thermo Fisher, MA) at a final dilution of 0.1× for an additional 30 min to characterize cell growth and matrix development, respectively, by confocal microscopy. To further characterize the biofilm matrix for the presence of extracellular DNA (eDNA) and lectins, the hanging drops were similarly stained with the viability stain SYTO 9 for 30 min, followed by 4′,6-diamidino-2-phenylindole (DAPI) (Thermo Fisher, MA) at a final concentration of 500 nM and then a wheat germ agglutinin-Texas Red conjugate (WGA) (Thermo Fisher, MA) at a final concentration of 50 μg/ml. In each case, 5 μl of the drop was sequentially replaced by the stain. Unbound stains in the resulting aggregates were rinsed twice by extracting 5 μl of the medium from the drops from the top of the drop and pipetting in 5 μl of fresh TSB, without disturbing the biofilms in the bottom of the drop. The aggregates were rinsed once more and carefully transferred with minimal disturbance to the underside of a coverslip sealed over a microscope slide with petroleum jelly. The biofilms of clinical isolates were stained according to the same protocol. One field of view per biofilm was imaged using a Zeiss LSM 700 upright confocal microscope (Carl Zeiss, NY) with an Apochromat 63× oil objective with a vertical stack interval of 1 μm.

### Quantification of hanging-drop biofilm morphology.

To quantify biofilm architecture, the confocal images were analyzed using Imaris v9.5 (Oxford Instruments, MA). The average intensities of SYTO 9 and SYPRO ruby in every image of the *z*-stack were obtained using an in-house MATLAB script (https://github.com/tcheng1124/Matlab.git) at various time points. These intensity values were then used to calculate the growth of the structure over time after applying a minimum threshold value. In addition, the distributions of microcolony sizes and volume colocalization statistics were obtained using the Imaris image-processing module. The microcolony size distribution was obtained using the surface-rendering feature using the green channel and a minimum volume threshold of 2.00 μm^3^. The Pearson correlation coefficient (PCC) was estimated using a built-in function in Imaris software.

### Dose response of hanging-drop biofilms to antibiotics.

The compounds ampicillin, vancomycin, tetracycline, and auranofin were all purchased from Sigma-Aldrich (St. Louis, MO). The stock solutions of ampicillin and vancomycin were prepared by dissolving the powdered solid in cell culture water, and stock solutions of tetracycline and auranofin were prepared by dissolving the drugs in dimethyl sulfoxide (DMSO) to a final concentration of 10 mg/ml. The working solutions were prepared by 10-fold serial dilution of the respective stock solutions in TSB to the final desired concentration. For biofilm inhibition assays, 5 μl of the drug solution diluted in fresh TSB medium was mixed with 45 μl of the cell suspension and placed as a hanging drop in the well plates. The hanging drops were analyzed after 24 h. For biofilm eradication assays, after 24 h of biofilm formation, 5 μl of the hanging drop was replaced with an equal volume of the drug solution diluted in fresh TSB medium and incubated for an additional 24 h. Five microliters of 10% bleach was used as a control treatment to model 1% antibiotic effectiveness for both inhibition and eradication assays. For analysis, the hanging drops were flushed with 5 μl of PrestoBlue (Thermo Fisher, MA) and allowed to incubate for 10 min. Afterward, the hanging drops were transferred to a GravityTRAP plate, and fluorescence was read at an excitation/emission wavelength of 530/590 nm. The antibiotic susceptibility of surface-attached biofilms was evaluated using both biofilm inhibition and eradication assays. For inhibition of biofilm formation, the cells were seeded with drugs in 96-well plates, and the activity was measured using a PrestoBlue assay after 24 h. For eradication of preformed biofilms, the biofilms formed after 24 h were freed of nonadherent cells by gently removing the medium and rinsing with PBS and then treated with drugs dissolved in medium for an additional 24 h.

### Formation, analysis, and dose response of surface-adhered biofilms to antibiotics.

TCH1516 was initially isolated from an adolescent patient with severe sepsis syndrome. Five hundred microliters of a TCH1516 cell suspension (final OD_600_ = 0.01) in TSB medium supplemented with 0.5% glucose and 5% human serum was added to a 4-well tissue culture-treated chamber slide (Lab-Tek; Thermo Fisher, MA) precoated with rat tail collagen type I (Corning; Fisher Scientific, PA) at a final concentration of 100 μg/ml. The samples were incubated for 24 h. For imaging of adherent biofilms, the medium was removed, nonadherent cells were removed by a gentle rinse with 100 μl of fresh TSB, and 100 μl of each stain was added at the same concentrations as the ones used for the adherent biofilms. The metabolic activity of surface-attached biofilm cultures was estimated using the PrestoBlue assay according to the manufacturer’s protocol.

For monitoring the growth of clinical isolates, untreated 96-well plates and TSB without any additives were used, in order to maintain consistency with clinical testing methods. Viability was assessed using a crystal violet assay without fixation, according to a previously reported protocol (45).

### QuantiGene Plex gene expression assay for select S. aureus genes.

The expression levels of the *arcB*, *ureC*, and *sdrC* genes, normalized with the *gmk* housekeeping gene, under different growth conditions were assessed using custom-designed QuantiGene Plex assays (S. aureus, catalog number M20111601; Affymetrix Inc., Santa Clara, CA) according to the manufacturer’s instructions (26). The results were expressed as fold increases in expression levels in biofilms over the expression levels in planktonic cells in mid-log phase. Briefly, the bacterial pellets were lysed using cold TES [*N*-tris(hydroxymethyl)methyl-2-aminoethanesulfonic acid] buffer with lysozyme and lysostaphin, followed by proteinase K (all from Sigma) as a homogenizing solution. Supernatants were isolated and incubated with working bead mix (capture beads) at 54°C for 18 h under gentle agitation at 600 rpm. After incubation overnight, the sample was transferred to a magnetic separation plate. Using a handheld magnetic plate washer, the samples were washed three times with wash buffer in between 1-h incubations at 50°C with preamplifier solution, amplifier solution, and label probe solution. The samples were then incubated with streptavidin-conjugated R-phycoerythrin (SAPE) working reagent for 30 min at room temperature, followed by 3 rinses with SAPE wash buffer. Each sample received 130 μl of SAPE wash buffer and was mixed at 800 rpm for 3 min. The plate was read on a Bio-Plex 200 system with Bio-Plex Manager software version 6.1, build 727 (Bio-Rad Laboratories Inc., Hercules, CA), and the gene expression profiles were analyzed.

### Statistical analysis.

For confocal microscopy, viability, metabolic activity, and dose-response experiments, multiple hanging-drop biofilms or surface-attached biofilms were collected. Each experiment consisted of at least three, but typically six, replicate biofilms, and the experiments were independently repeated on different days. For gene expression analyses, four replicate biofilms were pooled to obtain enough RNA, and this experiment was repeated three independent times. To establish statistical significance, unpaired Student’s *t* test was used, and for gene expression analysis, Dunnett’s multiple-comparison test was used. A *P* value of <0.05 was deemed a significant difference between groups.
