# Supplementary figures and images for: A Facile High-Throughput Model of Surface-Independent Staphylococcus aureus Biofilms by Spontaneous Aggregation
Source: mSphere. 2021 Apr 28;6(2):e00186-21. doi: 10.1128/mSphere.00186-21 (PMC8092136; doi:10.1128/mSphere.00186-21)

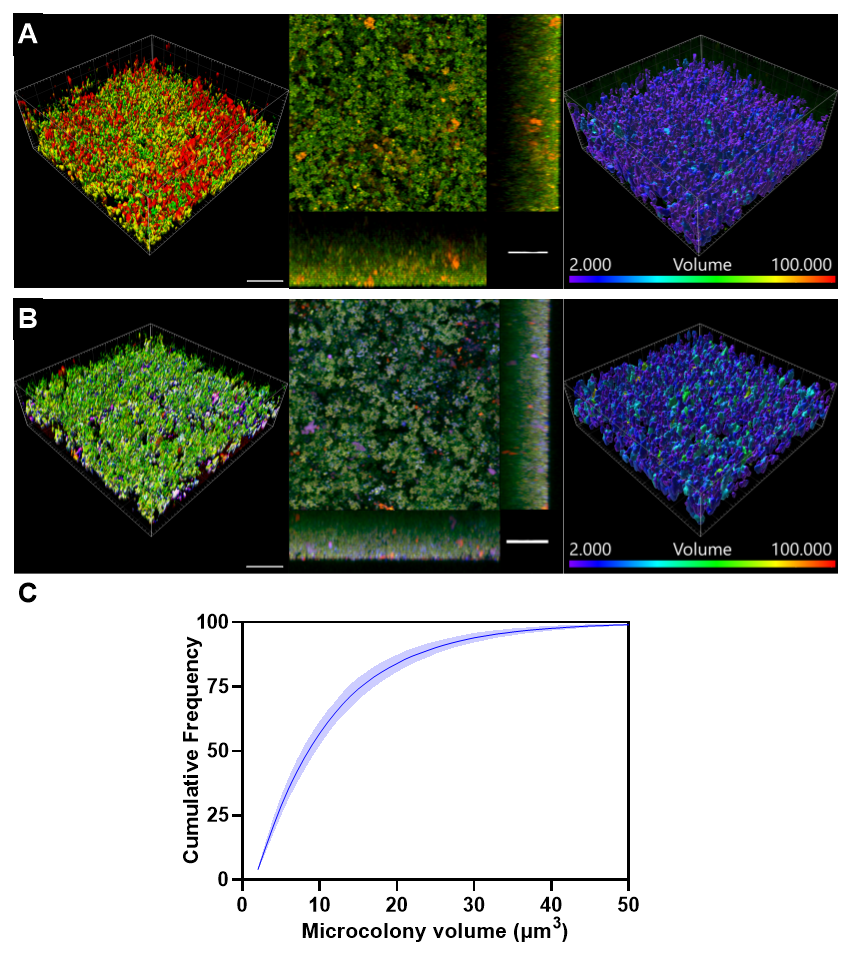

Supplement: FIG S1 [file mSphere.00186-21-sf001.tif]

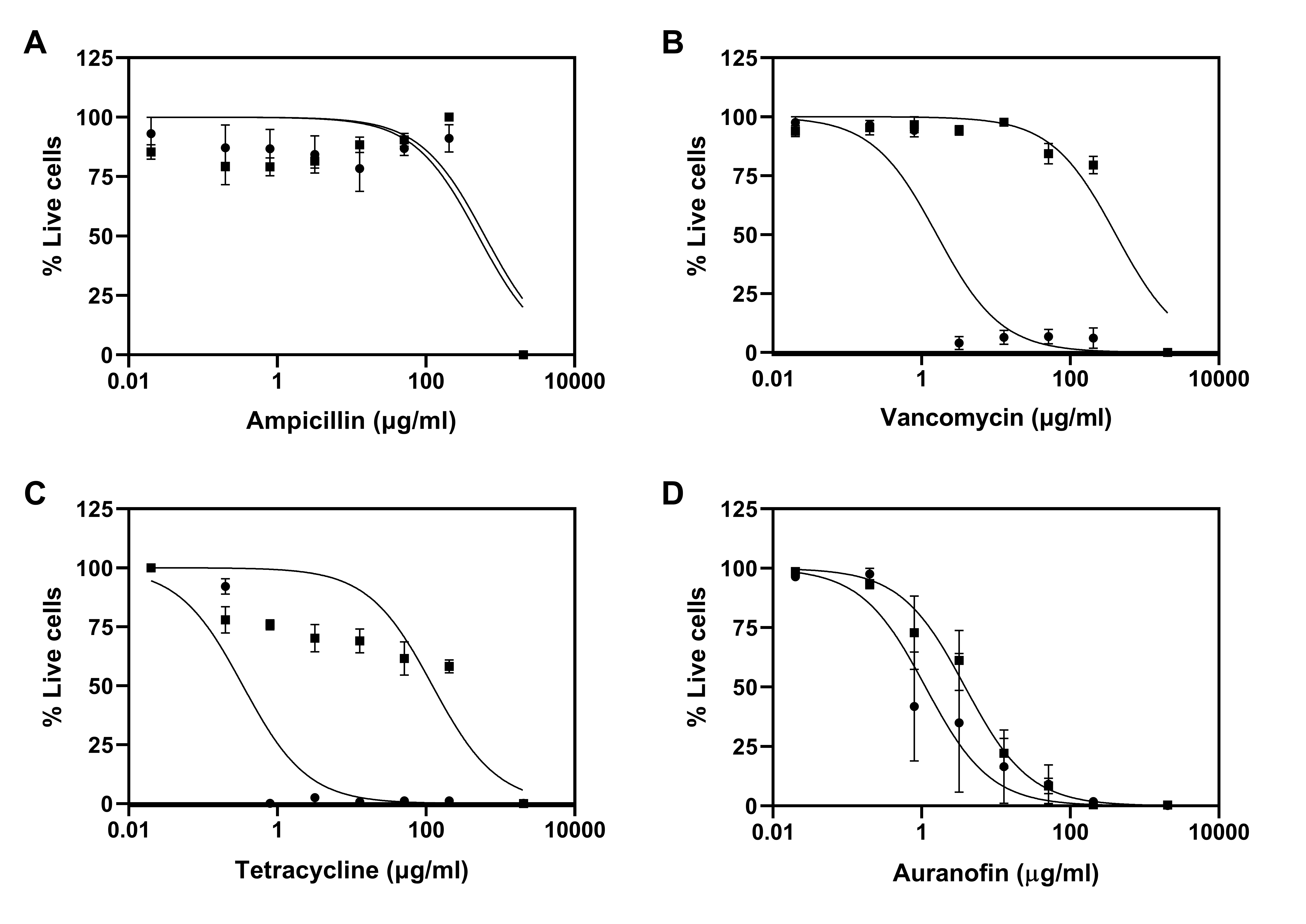

Supplement: FIG S2 [file mSphere.00186-21-sf002.tif]

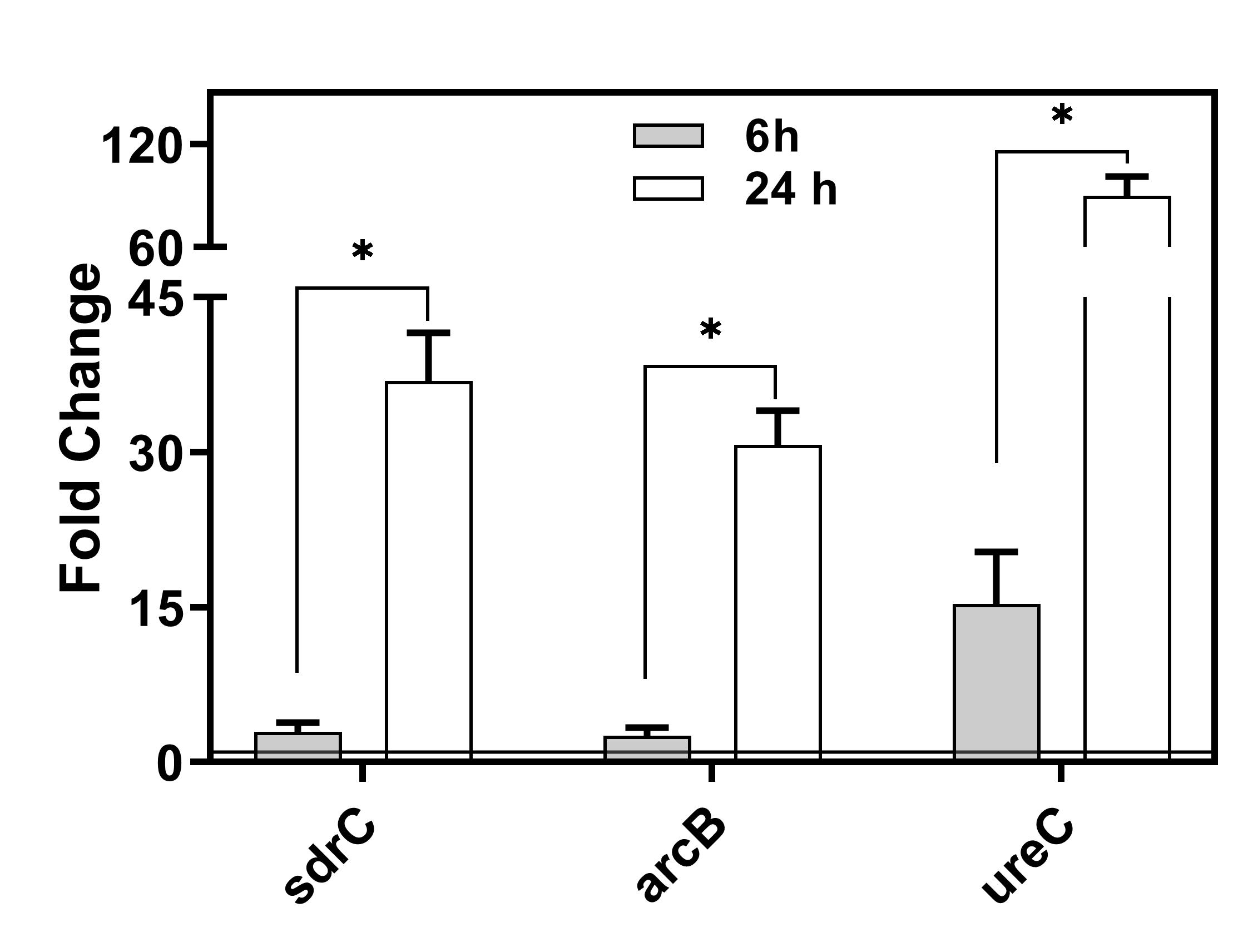

Supplement: FIG S3 [file mSphere.00186-21-sf003.tif]

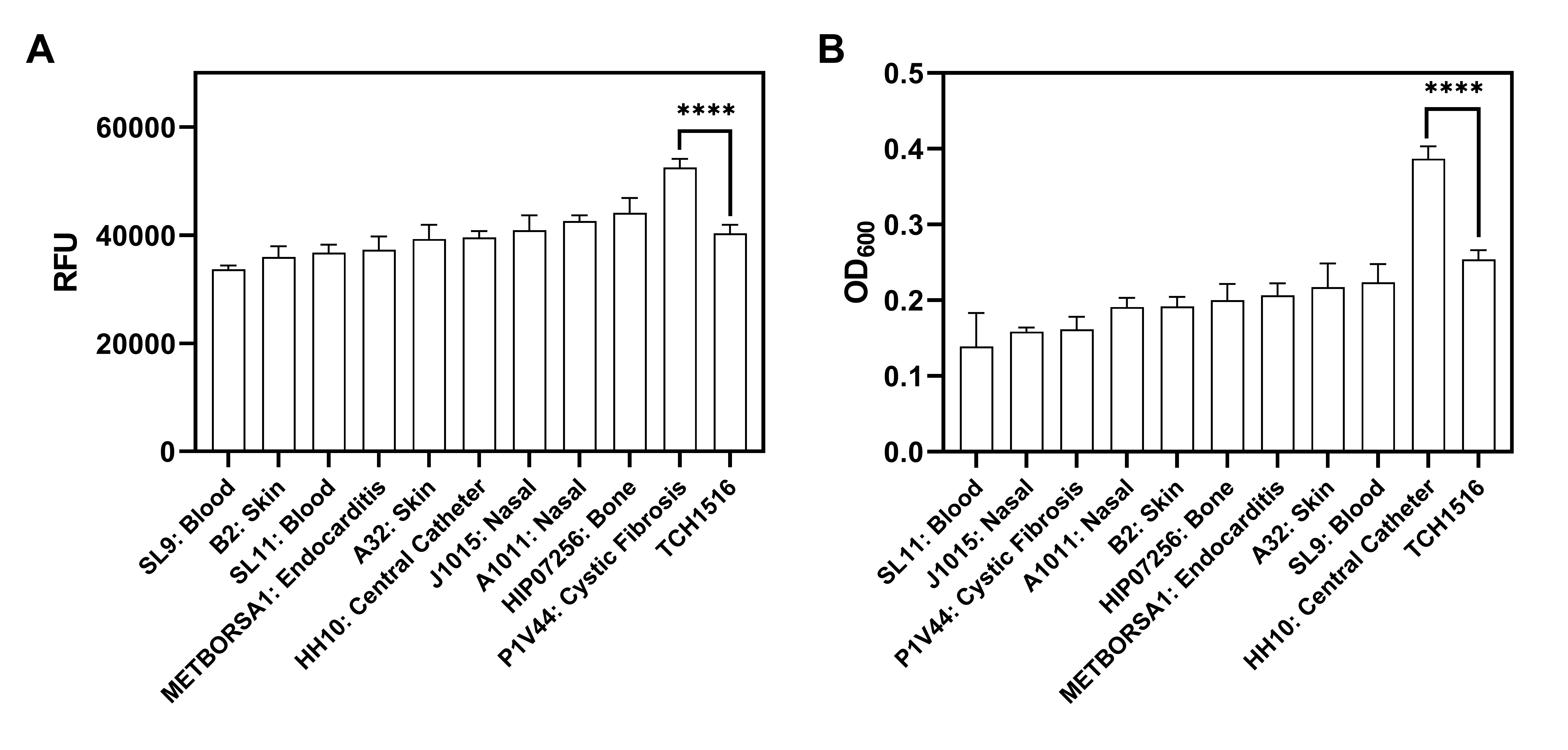

Supplement: FIG S4 [file mSphere.00186-21-sf004.tif]
